# Two novel pathway analysis methods based on a hierarchical model

**DOI:** 10.1093/bioinformatics/btt583

**Published:** 2013-10-11

**Authors:** Marina Evangelou, Frank Dudbridge, Lorenz Wernisch

**Affiliations:** ^1^Medical Research Council Biostatistics Unit, Institute of Public Health, Cambridge, CB2 0SR, UK, ^2^JDRF/Wellcome Trust Diabetes and Inflammation Laboratory, NIHR Cambridge Biomedical Research Centre, Cambridge Institute for Medical Research, University of Cambridge, Addenbrooke’s Hospital, Cambridge, CB2 0XY, UK and ^3^Faculty of Epidemiology and Population Health, London School of Hygiene and Tropical Medicine, London, WC1E 7HT, UK

## Abstract

**Motivation:** Over the past few years several pathway analysis methods have been proposed for exploring and enhancing the analysis of genome-wide association data. Hierarchical models have been advocated as a way to integrate SNP and pathway effects in the same model, but their computational complexity has prevented them being applied on a genome-wide scale to date.

**Methods:** We present two novel methods for identifying associated pathways. In the proposed hierarchical model, the SNP effects are analytically integrated out of the analysis, allowing computationally tractable model fitting to genome-wide data. The first method uses Bayes factors for calculating the effect of the pathways, whereas the second method uses a machine learning algorithm and adaptive lasso for finding a sparse solution of associated pathways.

**Results:** The performance of the proposed methods was explored on both simulated and real data. The results of the simulation study showed that the methods outperformed some well-established association methods: the commonly used Fisher’s method for combining *P*-values and also the recently published BGSA. The methods were applied to two genome-wide association study datasets that aimed to find the genetic structure of platelet function and body mass index, respectively. The results of the analyses replicated the results of previously published pathway analysis of these phenotypes but also identified novel pathways that are potentially involved.

**Availability**: An R package is under preparation. In the meantime, the scripts of the methods are available on request from the authors.

**Contact**: marina.evangelou@cimr.cam.ac.uk

**Supplementary Information**: Supplementary data are available at *Bioinformatics* online.

## 1 INTRODUCTION

It is increasingly recognized that pathway analysis can support exploration of genome-wide association study (GWAS) data by incorporating the available biological knowledge of genes and pathways. Pathway analysis has gained great popularity over the past few years and several methods have been proposed (Holmans *et al.*, 2009; O’Dushlaine *et al.*, 2009; Wang *et al.*, 2007; Yu *et al.*, 2009).

One of the first proposed and most popular pathway analysis methods for GWAS data is gene set enrichment analysis (GSEA). GSEA was originally proposed for pathway analysis of gene expression microarray data and later adjusted by Wang *et al.* (2007) for GWAS data. GSEA tests the null hypothesis that the pathway genes (or SNPs) are no more associated with the studied phenotype than the non-pathway genes (or SNPs), and is an enrichment method in the terminology of Goeman and Buhlmann (2007).

Other pathway analysis methods include Fisher’s method (FM): its test statistic depends on the product of the pathway gene (SNP) *P*-values. In addition, Yu *et al.* (2009) proposed the adaptive rank truncated product method (ARTP) for pathway analysis of GWAS data. The ARTP method is a generalization of FM based on the product of the most significant pathway gene (or SNP) *P*-values. The number of the smallest *P*-values multiplied is found through a permutation procedure.

In addition, a number of online bioinformatics tools have made their appearance over the recent years that test whether the pathway in question contains more significant genes than expected by chance, using either the hypergeometric test or Fisher’s exact test.

Most of the current pathway analysis methods are frequentist methods that depend on the results of single SNP analysis. In this article, two novel methods are proposed that are based on a hierarchical framework that models both pathway and SNP level effects. As discussed by Wang *et al.* (2010), hierarchical modelling is another form of pathway association testing. Wang *et al.* (2010) pointed out that hierarchical modelling and shrinkage techniques have not been widely used in pathway analysis. On the other hand, Bayesian hierarchical modelling is discussed as a powerful technique in decreasing the signal to noise ratio in GWAS and for handling the problems of multiple comparisons observed in several statistical applications, for example, analyzing GWAS data (Chen and Witte, 2007; Hung *et al.*, 2004; Liu *et al.*, 2005).

Few pathway analysis methods have been published that use hierarchical modelling for identifying associated pathways. Two are the BGSA method suggested by Shahbaba *et al.* (2012) and the method proposed by Wang *et al.* (2011). BGSA is based on a two-level hierarchical model, where in the first level the unsquared Cochran–Armitage statistics of the lead SNP of each gene are assumed to follow a normal distribution with mean zero. A scaled-inverse-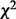 distribution is assumed for the variance of the SNP parameters. On the second level, a mixture of scaled-inverse-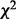 distributions is assumed for the variance of the gene parameters of each pathway. A *P*-value of association between the tested pathway and the phenotype is computed using Markov chain Monte Carlo.

Wang *et al.* (2011) proposed a mixed model that includes a fixed effects component that represents the mean disease contribution of a tested pathway and a random effects component that represents how the association of the pathway gene members with the disease varies around the pathway mean. As discussed by those authors, the proposed model allows information to be borrowed across genes in the same pathway. It further corrects for LD between SNPs and for the presence of overlapping genes.

In contrast to the methods proposed by Shahbaba *et al.* (2012) and Wang *et al.* (2011), the two methods proposed in this article use the individual genotype data and do not depend on the results of single SNP analysis. Furthermore, in contrast to other hierarchical models that incorporate either the SNP-pathway membership or the gene-pathway membership in the second stage of their multilevel models (Hung *et al.*, 2004; Lebrec *et al.*, 2009), our framework models hundreds of SNPs and pathways instead of a small selection of them.

Furthermore, our methods do not depend on Markov chain Monte Carlo techniques for identifying associated pathways. The two methods differ in the prior distribution assumed for the pathway parameters. The first method assumes a normal distribution for the pathway parameters with mean zero and diagonal variance–covariance matrix with equal entries. It uses Bayes factors to test whether the pathways are associated with the phenotype. The second method assumes a normal distribution with mean zero and a diagonal variance–covariance matrix whose entries are not necessarily equal. This method adapts an iterative algorithm proposed by Wipf and Nagarajan (2008) that uses an adaptive lasso algorithm that shrinks the diagonal entries towards zero. In this article, this algorithm is adapted for finding the set of associated pathways that explain the most variation of the studied phenotype.

First, the two proposed methods were applied to simulated data. Then, we applied the two proposed methods to two different GWAS studies that have been previously described by Evangelou *et al.* (2012). The first GWAS, which we refer to as the Platelets GWAS, aims to find the genetic structure of platelet function. These data were first presented by Jones *et al.* (2007, 2009). The second GWAS, which we refer to as the EPIC-Norfolk GWAS, aims to find the genetic structure of body mass index (BMI), as part of the EPIC-Norfolk study (Day *et al.*, 1999).

## 2 MATERIAL AND METHODS

### 2.1 Data

For the pathway analysis conducted in this article, 211 pathways from the Kyoto Encyclopaedia of Genes and Genomes (KEGG) were downloaded and tested (Kanehisa and Goto, 2000). The Platelets GWAS involves 500 individuals who took part in the studies of Jones *et al.* (2007, 2009). Four endpoints were measured for the 500 individuals: p-selectin and fibrinogen responses to ADP and collagen agonists, respectively. The individuals were also genotyped using the Illumina610 chip. By applying standard quality control filters, only 480 individuals were retained for further analysis. In addition, only SNPs with MAF >0.05, *P*-value of HWE >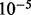 and proportion of missing values <0.05 were retained for further analysis. In total, 544 078 SNPs were retained for further analysis.

The EPIC-Norfolk GWAS involves 3559 individuals whose BMI was measured. The BMI ranges between 16 and 48, with a mean of 28.5. Individuals with a BMI >30 are defined to be obese. In the study, there are 2035 controls and 1514 obese cases. The BMI of three individuals was missing. The individuals were genotyped using the Affymetrix500 chip. By applying standard quality control filters on the SNPs, 382 037 SNPs were retained for further analysis.

For both studies, the SNPs of each study were assigned to genes according to physical distance: a SNP was mapped to every gene whose coding sequence had an overlap with a 10 kb range around the SNP.

### 2.2 Model

In contrast to single SNP analysis in which each SNP is tested independently from the others, in a multi-SNP analysis a selection of SNPs is tested for association. If the phenotype is a continuous random variable, the association between the phenotype and the *L* tested SNPs is modelled through a linear regression such that
(1)
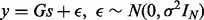

where *N* is the number of individuals in the study. The vector *s* of size *L* represents the SNP coefficients. The matrix *G* is the genotype matrix of size 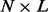 with entries 0, 1 and 2 representing the number of copies of the minor allele of each SNP. The error *ε* is assumed to follow a normal distribution with mean zero and variance 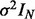.

The hierarchical model adds a second level of linear regressions for the SNP parameters *s*, which represent the pathway memberships of the tested SNPs. The SNP parameters *s* are assumed to follow the linear model
(2)
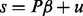

where *P* is a pathway matrix of size 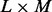, and *M* is the total number of pathways tested. The pathway matrix has entries 1 and 0 indicating whether a SNP belongs to a pathway. If a SNP was mapped to multiple genes, then this SNP was considered to be a member of all the pathways that the genes were members of. In addition, if a SNP was mapped to multiple genes that belonged to the same pathway, then no extra weight was given to these SNPs. The β vector of size *M* represents the pathway coefficients. The error *u* is assumed to follow a normal distribution with mean zero and variance 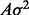. Here we assume for simplicity that the hyper-parameter matrix *A* is a diagonal matrix with equal entries along the diagonal, such that 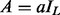 where *a* is a positive constant. This matrix assumes that all SNP effects come from the same distribution and that they are independent. Similarly to Guan and Stephens (2011), we preferred to model the SNP effects as independent, as they reflect the causal effects of the genotype matrix on the phenotype *y*, and they do not necessarily follow the same correlation structure as that of the SNP genotypes.

By combining Equations (1) and (2), the full model can be obtained
(3)
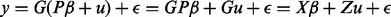

where *X* equals the genotype matrix (*Z* = *G*) multiplied by the pathway matrix (*P*). The random variables *ε* and *u* follow 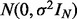 and 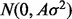 distributions, respectively.

The vector *u* represents the SNP parameters, which are treated as nuisance parameters and integrated out from the analysis. By conditioning on the pathway parameters β, the prior predictive distribution of 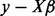 is a normal distribution with mean zero and covariance matrix 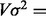 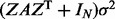. The probability density function of 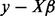 is given by
(4)
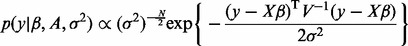



In other words, the phenotype *y* follows a normal distribution with mean 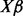 and variance 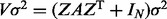.

Two prior distributions are considered for the pathway coefficients β. Each prior distribution is linked to a different procedure for identifying the associated pathways. We propose a separate inference method for each of the two prior distributions. The first method is based on Bayes factors and the second on the iterative algorithm of Wipf and Nagarajan (2008).

### 2.3 Normal/Bayes factors method

The first prior distribution considered is a normal distribution with mean zero and variance 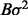. The hyper-parameter matrix *B* is a diagonal matrix with equal entries along the diagonal, such that 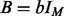, where *b* is a positive constant. The diagonal matrix assumes that pathway effects are independent and come from the same distribution.

By assuming a scaled-inverse-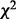 distribution with parameters 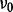 and 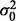 for the error variance 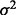, the association of pathways with the phenotype can be analytically calculated using Bayes factors (BFs).

The predictive likelihood of *y* conditioned on the variance components is a normal distribution with mean zero and covariance matrix
(5)
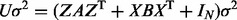

By integrating out the variance components, the marginal likelihood of *y* is a multivariate *t*-distribution with mean 0, degrees of freedom 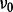 and variance 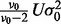 (Newton *et al.*, 2011).

The marginal distribution of *y* is used for testing the null hypothesis *H*_0_: the *q^th^* pathway is not associated. This null hypothesis is tested using BFs, the ratio of the probability of the data under a model without the pathway in question over the probability of the full model including all pathways. The logarithm of the BF for pathway *q* is given by
(6)
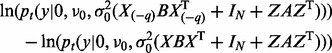

where 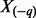 is the *X* matrix with the contribution of the *q^th^* pathway removed. The *p_t_* denotes the probability density function of a random variable with a multivariate *t*-distribution with 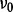 degrees of freedom, mean equal to zero and variance 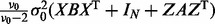 under the null model. The smaller the value of the BF, the greater the evidence that the pathway should be kept in the model and the hypothesis *H*_0_ rejected.

### 2.4 Sparse normal/Adaptive lasso

The second prior distribution assumes that the pathway effects do not necessarily have the same variance and it allows some of variances to be zero. The prior distribution is a normal distribution with mean zero and variance Γ, where Γ is a diagonal matrix with positive or zero entries along the diagonal. The associated pathways are found using the iterative algorithm proposed by Wipf and Nagarajan (2008), which solves an adaptive lasso problem in each iteration. The adaptive lasso, like the standard lasso (Tibshirani, 1996), shrinks most of the pathway coefficients to zero resulting in a sparse solution with only the pathways that are significantly associated with the tested phenotype having non-zero coefficients.

Wipf and Nagarajan (2008) proposed an algorithm that finds the solution of the sparse regression problem using automatic relevance determination (ARD)
(7)
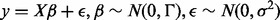

where the hyper-parameter matrix Γ is a diagonal matrix with positive or zero entries along the diagonal.

The diagonal entries of Γ can be estimated from the data by first marginalizing over the coefficients β and then performing a type II maximum likelihood, which is equivalent to minimizing
(8)
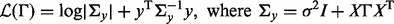

where a flat hyper-prior is assumed for the diagonal entries of Γ. The associated pathways correspond to the non-zero entries of Γ. A full description of the iterative algorithm can be found in Wipf and Nagarajan (2008). Compared with other EM-type or fixed point algorithms that have been proposed for minimizing Equation (8), their algorithm is less prone to get stuck in a local minimum.

In each iteration of the algorithm, an adaptive lasso problem is solved. An adaptive lasso problem is similar to the original lasso problem with the difference that individual adaptive weights are assigned to each parameter. The weights of the adaptive lasso problem are updated in each step of the algorithm of Wipf and Nagarajan (2008). The adaptive lasso problem can be solved using the least angle regression procedure as suggested by Zou (2006).

The probability density function presented in Equation (4) can be formulated as in Equation (7) by rewriting it as

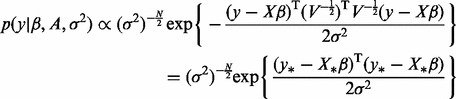

The vector 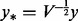 follows a normal distribution with mean 
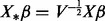
 and variance 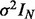, where the matrix *V* equals 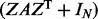 as seen earlier. A square root of the matrix 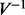 can be calculated, for example, by its Cholesky decomposition. A sparse normal prior distribution with mean zero and variance Γ is assumed for the β coefficients, where Γ is a square diagonal matrix.

The iterative algorithm proposed by Wipf and Nagarajan (2008) can therefore be applied to find a sparse solution for
(9)
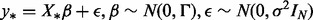



The error variance 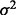 acts as the tuning parameter that controls the shrinkage of the ARD. The smaller the value of 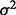, the greater the shrinkage applied; therefore, fewer diagonal entries of Γ are non-zero. The non-zero diagonal entries of Γ correspond to non-zero β coefficients and associated pathways.

## 3 SIMULATION STUDY

The proposed methods were compared in a simulation study with FM and BGSA (Shahbaba *et al.*, 2012). As discussed by Evangelou *et al.* (2012), FM is one of the most powerful enrichment and association methods for pathway analysis. The FM statistic is twice the negative of the logarithm of the product of the SNP *P*-values within each pathway. Its significance is calculated using a 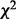 distribution with degrees of freedom equal to twice the number of SNPs in the pathway. An additional aim of the simulation study was to find the appropriate hyper-parameters for the two proposed methods.

To design realistic simulation models, the Platelets GWAS data were used. Both the genotype and pathway matrices include 90 061 SNPs in 4767 genes that are members of the 211 KEGG pathways. For simplicity only the SNPs with no missing values were tested in the simulation study, leaving 50 254 SNPs for inclusion.

The simulated phenotypes were computed as
(10)
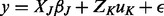

where *X* and β are restricted to a subset of *J* selected pathways, and *Z* and *u* are restricted to a subset of *K* selected SNPs. The selected SNPs and pathways were the only SNPs and pathways assumed to have a non-zero effect on the simulated phenotypes. These SNPs and pathways were selected from analyses of the p-selectin response to collagen (PC) phenotype in the Platelets GWAS, as described later in text. The variance of *ε* is set to the residual error of the linear regression given in Equation (10) when fitted to real data. The pathway and SNP parameters were also found through Equation (10) when fitted to real data.

We chose the first 100 SNPs with the smallest individual *P*-values of association with the PC phenotype as the subset of *K* selected SNPs. The *P*-values of these SNPs ranged from 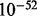 to 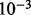.

In the first scenario of the simulation study, the *J* associated pathways were selected using the Normal/Bayes factors method (NBF) method. Two different hyper-parameter combinations were tested. The hyper-parameter combinations used are 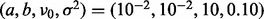 and 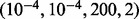, respectively. The selected pathways in both cases had BFs ≤1. The number of associated pathways for the two sets of hyperparameters was 24 and 8, respectively.

In the second scenario of the simulation study, the *J* associated pathways were found using the Sparse normal/Adaptive lasso (SNAL) method. In the first case of the second scenario, the hyper-parameter matrix factor *a* was set to 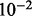 and the tuning parameter 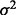 to 0.05, whereas in the second case *a* was 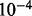 and 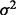 0.5. Applying this model to the Platelets GWAS, 24 and 15 pathways were selected to have effects on the simulated phenotypes in the two cases.

Two more scenarios were simulated. In the third scenario, two cases were simulated in which the selected pathways had FM *P*-values <0.05 and < 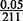, which resulted in 39 and 10 pathways, respectively. For both cases, the largest pathway (which contains 9397 SNPs) was considered to be associated, and it contained 19 associated SNPs. This is the largest number of associated SNPs contained in a single pathway.

Similarly, in the fourth scenario, two cases were simulated. In the first case, the selected pathways are the 201 pathways that had BGSA *P*-values <0.05 and in the second case the selected pathways are 8 pathways with BGSA *P*-values < 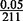.

A scaled-inverse-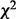 distribution with parameters (2,1) was assumed for the SNP parameters *u*. This is in contrast to Shahbaba *et al.* (2012), who considered an improper prior in their simulation study. This prior cannot be used here because there are genes in our dataset that had only two SNPs assigned to them. Shahbaba *et al.* (2012) in their simulation study, excluded pathways that had common genes and considered only genes that had >3 SNPs assigned to them. On the other hand, we used the same priors (and hyper-parameters) as the ones used in the simulation study of Shahbaba *et al.* (2012) for the pathway level statistics.

The performance of the methods was assessed by plotting receiver operator characteristic (ROC) curves and calculating the area under the ROC curves (AUC) (Fawcett, 2006). An ROC curve is a graphical plot of sensitivity against specificity. Both sensitivity and specificity were recorded for a range of BF thresholds, 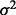 values and *P*-values. This enabled assessment of the overall power of the methods without assuming specific thresholds. Sensitivity is the true positive rate calculated as the ratio of pathways found to be associated divided by the number of true associated pathways. Specificity is one minus the false positive rate (FPR), which is the ratio of false positive pathways over the number of true non-associated pathways. The AUC represents the probability that a random true associated pathway has a lower score (*i.e. P*-value or BF) than a random non-associated pathway. An AUC close to 1 indicates an optimal method, whereas an AUC of 0.5 just random performance. The AUC is calculated using the convex hull of the ROC curve (Fawcett, 2006). Under each of the scenarios described, 50 simulated datasets were generated from the model in Equation (10) and the AUCs of each of the methods were estimated.

For NBF, a BF cut-off value has to be applied for finding the pathways that are associated with the response; a set of values between 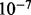 and 1 was considered as possible cut-off values. As the cut-off value increases, the number of associated pathways increases as does the number of false positives. For the SNAL method, the tuning parameter 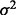 also took values between 1 and 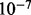. The smaller the value of 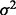, the smaller the shrinkage effect, *i.e.* more pathways are found to be associated. Further, for both FM and BGSA, the *P*-value cut-offs ranged between 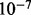 and 1. All sets of BF thresholds, 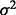 values and *P*-values were of size 50.

A number of different hyper-parameters were tested for the two proposed methods. The values of the hyper-parameter values *a* and *b* of the variance matrices *A* and *B* tested were 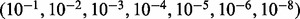. The hyper-parameter values of the scaled-inverse-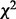 distribution were (10, 25, 50, 100, 200) for 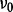 and (0.25, 0.50, 1, 2) for 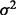. For each simulated phenotype, the combination of hyper-parameters that gave the highest AUC of the proposed framework was recorded.

## 4 RESULTS

### 4.1 Simulation study

The performance of the methods was tested on the simulated responses created under the four tested scenarios. Table 1 gives the mean and median AUCs for methods SNAL, NBF, FM and BGSA across the four tested scenarios. As can be seen from the table, SNAL and NBF perform better than both FM and BGSA, with SNAL performing slightly better than NBF. The differences between the two proposed methods and the FM and BGSA methods are statistically significant. Using paired *t*-tests, the four methods were compared. FM is statistically superior to BGSA with a *P*-value of order 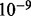. Similarly SNAL and NBF are statistically superior to FM, with *P*< 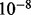. SNAL is statistically superior to NBF with a *P*-value <0.05 (*P* = 0.0043). The mean and median AUCs of the four methods for each one of the tested scenarios (and their subcases) are given in the Supplementary Tables S3–S6.

**Table 1.** Mean and median AUCs (with their standard deviations in parentheses) of the four tested methods across the four tested scenarios of the simulation study

Method	Mean AUC	Median AUC
SNAL	0.7551 (0.1543)	0.8050 (0.1948)
NBF	0.7469 (0.1665)	0.7888 (0.2400)
FM	0.6971 (0.0644)	0.6932 (0.0766)
BGSA	0.6576 (0.1456)	0.6121 (0.1091)

As discussed in the previous section, the hyper-parameters with the highest AUCs of the methods were recorded. The hyper-parameters that achieved the maximum AUC most frequently across all 50 simulations of phenotypes for each of the four tested scenarios are 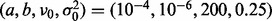 for NBF and 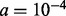 for SNAL.

**Fig. 1.**
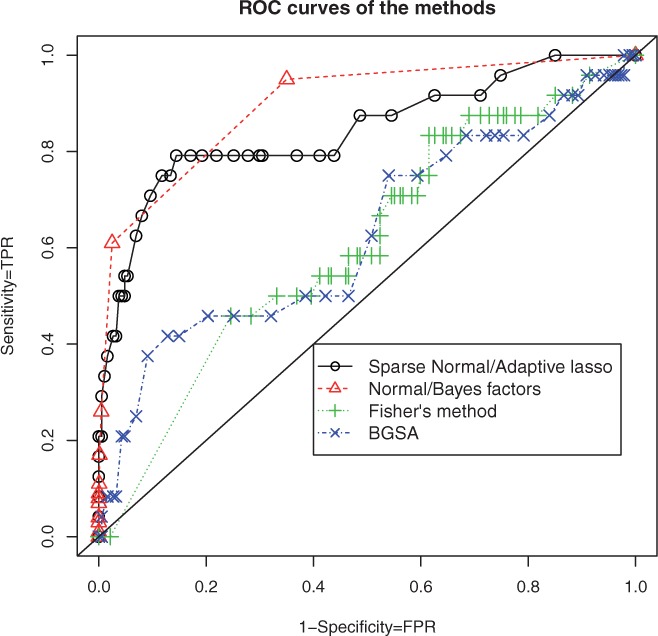
ROC curves of the four methods for different simulated responses. The hyper-parameter combination of the NBF method is 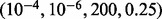. The hyper-parameter 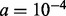 for the SNAL method. The simulated response was created in the first case of the second scenario, *i.e.* the selected pathways were selected by SNAL

Figure 1 shows the ROC curves of the four tested methods for simulated responses generated in the first case of the second scenario. The selected pathways were chosen by SNAL with the hyper-parameter 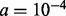 and the tuning parameter 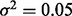. The plot includes the ROC curves of the four tested methods and a diagonal line that represents the random model that classifies a pathway as associated with a probability equal to 0.50. The further away an ROC curve is from the diagonal line, the better the performance of the method is. As can be seen, the ROC curves of FM and BGSA are close to the diagonal line, whereas the ROC curves of the SNAL and of the NBF methods are further away. The proposed Bayesian hierarchical framework attains higher sensitivity compared with the other methods, whereas its specificity is at the same level.

For a BF cut-off of 1, the FPR of the NBF method is ∼0.30, whereas its true positive rate is ∼0.90. For an FPR ∼0.05, the sensitivity of NBF is slightly higher than that of SNAL.

Differences across the selected pathways chosen by the FM, SNAL and NBF methods were also observed. The average number of SNPs within a KEGG pathway is equal to 723 SNPs, whereas the average number of SNPs within a pathway from the ones that FM selected was 1252 (for the first case of scenario 3) and 1854 (for the second case of scenario 3). This phenomenon was not observed for either NBF or SNAL, where the average number of SNPs in the selected pathways was ∼500 SNPs.

Therefore, the performance of the four methods was further tested for another scenario. The selected *J* pathways were random pathways that either had <300 SNPs or >700 SNPs. Table 2 shows the performance of the methods for the two cases. As can be seen, the performance of FM drops significantly as the pathway size decreases. In addition, BGSA is also affected by the pathway size but less so than FM. On the other hand, both the NBF and SNAL methods are less affected. Paired *t*-tests were used to test the differences between the four methods for each one of the two cases. FM is statistically superior to all three other methods for the second case with *P*-values <0.05. On the other hand, for the first case, both NBF and SNAL are significantly different from the other two methods with *P*< 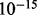. Unpaired *t*-tests were used to compare the differences of each method for the two cases. Only the differences of SNAL were not found significant, whereas for the other three methods differences between the two cases were statistically significant.

**Table 2.** Mean and median AUCs (with their standard deviations in parentheses) of the four methods

Random/Pathway size	Method	Mean AUC	Median AUC
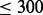	NBF	0.5891 (0.0265)	0.5843 (0.0155)
	SNAL	0.5940 (0.0388)	0.5925 (0.0420)
	FM	0.5074 (0.0147)	0.5000 (0.0000)
	BGSA	0.5197 (0.0232)	0.5065 (0.0097)
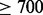	NBF	0.6094 (0.0431)	0.6101 (0.0400)
	SNAL	0.5803 (0.0472)	0.5667 (0.0367)
	FM	0.6692 (0.0439)	0.6755 (0.0440)
	BGSA	0.5553 (0.0295)	0.5556 (0.0315)

*Note*: The selected pathways correspond to random pathways that either contain <300 SNPs or >700 SNPs.

SNAL generally outperforms the other methods, followed by NBF. In addition, SNAL was not affected by the sizes of the pathways, whereas the other three methods were found to be affected by pathway size. FM had the greatest power in identifying pathways that were considered to be large compared with pathways that were smaller in size.

### 4.2 Platelets GWAS

The proposed Bayesian hierarchical framework was applied to the Platelets GWAS. It was applied to find pathways associated with the four endpoints that measure platelet function: p-selectin and fibrinogen responses to both collagen and ADP agonists as described by Jones *et al.* (2007, 2009).

As in the simulation study, the 211 KEGG pathways were tested. The SNPs of the study were mapped to the pathways. Only 90 061 SNPs of the study are members of the 211 KEGG pathways. Any missing genotypes were replaced by 0, 1 or 2 using mean imputation. The frequencies of genotypes 0, 1 and 2 were calculated and any missing genotypes were replaced by 0, 1 or 2 with probabilities equal to their frequencies.

Following the aforementioned simulation study, thresholds were chosen to give an FPR of 0.05. The NBF hyper-parameters used are 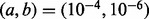 and 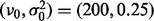, and for the SNAL method the hyper-parameter *a* equals 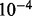.

The associated pathways identified by NBF are the ones with BFs ≤0.95. The tuning parameter 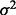 of the SNAL method was set equal to 0.5. The results of applying the methods on the four phenotypes of platelet function can be found in the Supplementary Tables S8–S11.

The KEGG pathways with the smallest BFs are Phagosome for PC, Tight junction for PA, Salivary secretion for FC and Leucocyte transendothelial migration for FA. There are a number of pathways that were found to be associated with >1 phenotype. For example, the Viral myocarditis pathway was found to be associated with the PC and FC phenotypes. In addition, the Melanogenesis pathway was found to be associated with PC, PA and FC phenotypes. Tight junction was further found to be associated with both PA and FA phenotypes.

SNAL identified on average, a smaller number of associated pathways than the NBF method. Similarly to NBF, SNAL identified pathways that were associated with at least two of the phenotypes. For example, the Glycolysis/Glycogenesis pathway as associated with PA and FA phenotypes, the pathway Vitamin b6 as associated with PC and PA phenotypes. In addition, the pathways of Glycosaminoglycan degradation and Glycosphingolipid biosynthesis were found to be associated with PC and FC.

There are pathways that were identified by both methods as associated. The five pathways identified by SNAL as associated with FA phenotype, were all included in the list of pathways with BFs <0.95. For the other three phenotypes, the proportions of pathways found by SNAL and NBF are >66%.

Basal cell carcinoma was found to be associated with phenotypes FA, FC and PC, and Renal cell carcinoma was found to be associated with phenotype FC. Basal cell carcinoma and Renal cell carcinoma pathways were found to be associated with all four endpoints by either method, either by both of the methods or by one of them.

### 4.3 EPIC-Norfolk GWAS

The pathway matrix of the EPIC-Norfolk GWAS has size 59 327 × 211. Any missing genotype values were imputed using mean imputation as the number of missing genotype values was small. Similarly with the Platelets GWAS, a simulation study was performed for identifying the hyper-parameters of the two methods that resulted in an FDR of 0.05 in the simulation. The appropriate hyper-parameter combination for the NBF method is 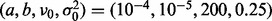 and 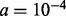 for the SNAL method. Pathways with BFs <0.765 were considered to be associated, and the tuning parameter 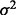 was set equal to 1.4.

NBF identified 18 pathways as associated with BMI, whereas SNAL identified 55 pathways (Supplementary Table S12). Amongst them 17 of these pathways were identified by both methods. Some of the pathways found to be associated with BMI are Cell cycle, Steroid biosynthesis, SNARE interactions in vesicular transport, Viral myocarditis, Fc epsilon RI signalling pathway, TGF-β pathway, Haematopoietic cell lineage, Glioma, Melanogenesis and Jak-STAT signalling pathway. Pathways related with glycolysis were also identified as, for example, Glycolysis/Gluconeogenesis, Other glycan degradation, Glycosaminoglycan biosynthesis and Glycerolipid metabolism.

The KEGG Haematopoietic cell lineage identified by Liu *et al.* (2010) as associated was found associated by both SNAL and NBF. The KEGG pathways Type 2 diabetes mellitus, PPAR signalling pathway, Fc epsilon RI signalling pathway identified by either or both NBF and SNAL as associated are members of the list of pathways related to obesity published by Park *et al.* (2011).

Amongst the pathways not reported previously for association with BMI or obesity are KEGG SNARE interactions in vesicular transport and KEGG Jak-STAT signalling pathway. SNARE interactions are biologically related with the adipocyte as a secretory organ, as was discussed by Bradley *et al.* (2001).

Furthermore, obesity was analyzed as a binary phenotype, with 1 if BMI was >30 and 0 if BMI was ≤30. Both NBF and SNAL were applied to the binary version of BMI, retaining the linear model for the purpose of testing association. The results of this application are presented in Supplementary Table S13. The associated pathway with the smallest BF is KEGG Jak-STAT signalling pathway; surprisingly this is not the reported pathway with the strongest association when BMI was analyzed as a quantitative random variable. Most of the KEGG pathways identified were also identified when the quantitative BMI was analyzed. The KEGG pathways not reported previously are ErbB signalling pathway, Gap junction, Alzheimer’s disease and Shigellosis.

The performance of the four methods for analyzing binary data was also tested through a simulation study. The results of this study are given in Supplementary Table S7.

## 5 IDENTIFICATION OF ASSOCIATED SNPs

Although our emphasis is on identifying associated pathways, our methods can also be applied to improve detection of associated SNPs while taking pathway membership into account. As was discussed in Section 2, the SNP parameters are treated as nuisance parameters and are integrated out of the analysis, for computing the pathway effects (β). Alternatively, the pathway effects can be considered as nuisance parameters and integrated out of the model given in Equation (3). By integrating out the pathway effects, the SNPs associated with the tested phenotype can be identified.

The two proposed methods can be used to identify the associated SNPs. For example, the SNP BFs of NBF can be computed as
(11)
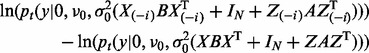

where 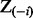 equals the genotype matrix with its *i^th^* column (*i.e.* the contribution of the *i^th^* SNP) removed. The 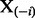 matrix equals the 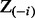 times the pathway matrix (*P*) with the contribution of its *i^th^* SNP (row) removed. The difference between a SNP BF and a pathway BF is that now the effect of the SNP is removed from both the genotype and pathway matrices.

NBF was applied to identify SNPs associated with platelet function. The results of the analysis are in agreement with previously published results that identified the GP6 gene as strongly associated with platelet function (Jones *et al.*, 2007, 2009). No other SNPs were identified as significantly associated.

NBF was also applied to the EPIC-Norfolk GWAS for identifying associated SNPs. The five SNPs with the smallest BFs are rs12488868, rs2952271, rs3393, rs8076465 and rs10887578. SNP rs12488868 lies close to ROBO1 gene on chromosome 3, the second and fourth SNPs lie on chromosome 17 close to gene PRKAR1A and SNP rs10887578 lies close to GRID1 gene (chromosome 10). All of the aforementioned genes have been reported as associated with BMI. SNP rs3393 lies close to ADORA3 gene (chromosome 1) and that has not been reported previously as associated with BMI. Gene ADORA3 is an adenosine A3 receptor involved in a number of intracellular signalling pathways and physiological functions. It is related with both neuroprotective and neurodegenerative effects as well as with cell proliferation and death.

## 6 DISCUSSION

In this article, a Bayesian hierarchical framework is proposed that incorporates pathway membership of SNPs through a two-level hierarchical model for identifying pathways associated with a phenotype. The phenotype of an individual is modelled to depend on both, its genotype and on a common contribution from alleles in each pathway. The SNP effects, over the common contribution from pathways they are members of, are assumed to be distributed identically and independently. These SNP effects are considered nuisance parameters and integrated out of the analysis so that the pathways associated with the disease can be identified. Intuitively, as is typical for random effects models, integration over SNP effect implies a correlation structure in the noise that is dependent on genotypes: individuals with similar genotypes show similar deviations from expected values of the regression model.

Within this hierarchical setting, we proposed two methods for selecting pathways as predictor variables for the phenotype. The NBF and the SNAL methods test the predictive power of a pathway, and hence its association with the phenotype. NBF uses the BF of the model that drops the pathway of interest over the full model that includes all pathways [see Equation (6)]. An alternative way to test for association was to compute the BF of the model with no pathways included over the one that includes the pathway of interest, which shows similar, slightly inferior, performance as FM (data not shown) and has not been further considered here. Alternatively, a full model selection with a combinatorial search over all possible sets of pathways could be performed but would be computationally demanding and has also not been considered here.

On the other hand, SNAL balances sparsity of predictors with explanatory power. The iterative algorithm proposed by Wipf and Nagarajan (2008), as used in SNAL, applies ARD, which achieves sparsity that lies between that achieved by *l*_0_ and *l*_1_ norm regularization [see Wipf and Nagarajan (2008) for details]. That is, it provides sparser results than *l*_1_ norm regularization (as implemented, e.g. by the lasso), but is not as computationally demanding as *l*_0_ norm regularization, which requires a combinatorial search through all sets of predictors. Therefore, SNAL provides the best compromise between the ideal of a systematic combinatorial search for the best set of pathways and computational feasibility.

The simulation study showed that both SNAL and NBF are able to identify pathways associated with the phenotype. Further, the performance of NBF improves with increasing pathway size, whereas that of SNAL is unaffected by the size of the pathways. This can be explained by the fact that some of the smaller pathways are subsets of larger pathways. Therefore, the predictors of the model are correlated, and NBF finds sparser solutions with good predictive behaviour by dropping some of these pathways.

NBF has the advantage over SNAL to be easily parallelized. The computing time of SNAL crucially depends on the algorithm used for a subroutine solving an *l*_1_ norm regularization problem. We used the least angle regression algorithm in this study. Depending on the number of predictors, other algorithms, for example, based on coordinate descents (Friedman *et al.*, 2010), could perform well.

The two methods were applied to the data of two GWAS on the genetic structure of platelets function and body mass index. Through simulation, suitable hyper-parameters of NBF and SNAL were found, as well as the appropriate BF cut-off and tuning parameter 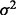 that gave an FPR of 0.05. As we pointed out in Section 2.4, the smaller the value of 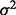 the more shrinkage is applied and the fewer associated pathways are found. The shrinkage parameter 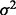 of SNAL, when applied to the BMI dataset, was found to be 1.4, larger than the one of the Platelets GWAS of 0.5. As discussed earlier, this is probably due to the variation of the studied phenotype and the number of correlated variables within the model. SNAL identified more pathways for BMI than NBF, whereas NBF found more pathways for platelet function than SNAL.

The results of the simulations showed that the methods outperform both FM and BGSA. BGSA is one of the recently published methods for pathway analysis that uses hierarchical modelling for identifying associated pathways. Shahbaba *et al.* (2012) compared BGSA with the well-known pathway enrichment methods ALIGATOR (Holmans *et al.*, 2009) and GSEA. The results of their analysis showed that the BGSA outperforms both methods.

In addition, we have recently published a comparison of current pathway analysis methods (Evangelou *et al.*, 2012). Three well-known association methods were adapted to test the competitive null hypothesis that the pathway genes are no more associated than the non-pathway genes. The three adapted association methods, tail strength measure, FM and ARTP, were compared with competitive methods, hypergeometric test and GSEA. The results of the analysis suggested that ARTP and FM should be preferred for both competitive and association testing. It should be noted that association methods have in principle better performance than competitive methods, as their tested null hypothesis is more stringent (Evangelou *et al.*, 2012). These results led us to compare our new methods with FM and BGSA.

Although the proposed methods are formulated in a Bayesian framework, an advantage is their implementation without time-consuming Monte Carlo simulation and their ability to deal with both large numbers of tested SNPs and pathways. They are also easily adapted to include other functional information on the SNPs in the design matrix *P*. This is comparable with the work of Chen and Witte (2007) but with the difference that our methods can handle larger numbers of SNPs and any individual level covariates. This is potentially important when dealing with structured populations or when allowing for environmental exposures.

We have considered a two-level model with SNPs assigned to pathways, but further levels of grouping SNPs, for example in genes, can be easily included potentially improving power by borrowing more information. This approach might be useful when dealing with rare variants that individually contribute little information. Further extensions of our approach are possible to allow for structure within the pathway database, such as the hierarchical organization of the Gene Ontology (The Gene Ontology Consortium, 2000). Finally, proper modelling of non-linear responses, including ascertained case/control status, will be an important future direction, although we believe that linear modelling yields reasonable power for the small genetic effects typically seen for complex traits.

## Supplementary Material

Supplementary Data
