# Supplementary material for: Two novel pathway analysis methods based on a hierarchical model
Source: Bioinformatics. 2013 Oct 11;30(5):690–7. doi: 10.1093/bioinformatics/btt583 (PMC3933872; doi:10.1093/bioinformatics/btt583)
Supplement: Supplementary Data [file supp_30_5_690__index.html]

Two novel pathway analysis methods based on a hierarchical model — Two novel pathway analysis methods based on a hierarchical model — Two novel pathway analysis methods based on a hierarchical model — Supplementary Data 

# Two novel pathway analysis methods based on a hierarchical model

## Supplementary Data

files

**Files in this Data Supplement:**

- Supplementary Data - pdf file
