# Supplementary material for: Two novel pathway analysis methods based on a hierarchical model
Source: Bioinformatics. 2013 Oct 11;30(5):690–7. doi: 10.1093/bioinformatics/btt583 (PMC3933872; doi:10.1093/bioinformatics/btt583)
Supplement: Supplementary Data [file supp_btt583_marinaevangelou-supplementary.pdf]

1 Model Checking

1.1 The effect of the hyper-parameters on the proposed methods

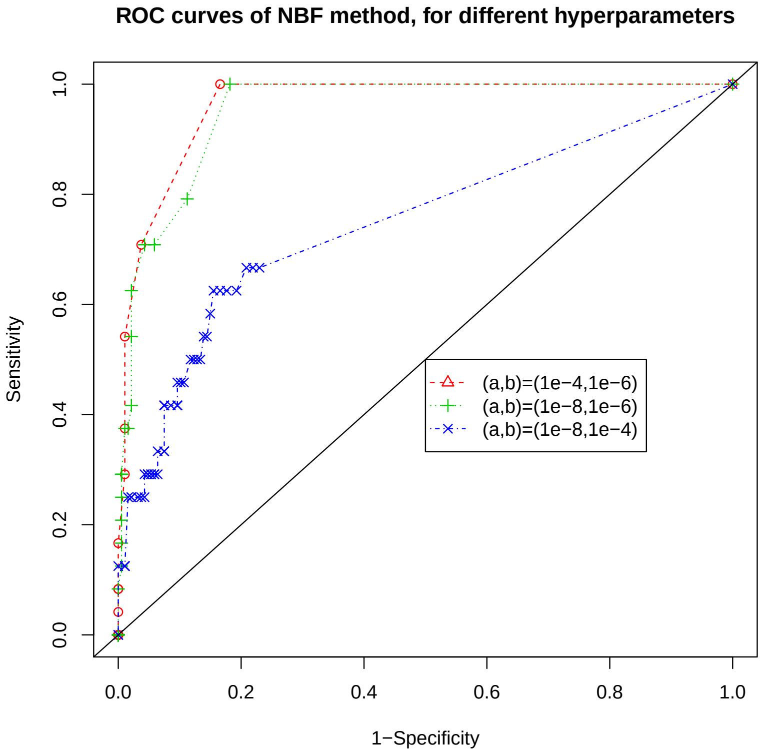

Figure 1: ROC curves of NBF for different hyper-parameters a and b. The AUC of the ROC curves for the three hyper-parameter combinations are 0.9643, 0.9596 and 0.7585, respectively. The hyper-parameters of the Inverse-Scaled-χ^2 distribution are $\nu_0 = 200$ and $\sigma_0^2 = 0.25$ for all three cases. The simulated response was created in the first case of the second scenario, ı.e the selected pathways were selected by SNAL.

Figures 1 and 2 show the effect of different hyper-parameters on the performance of the two proposed methods. As can seen, for all three different hyper-parameters a the performance of SNAL is very similar. On the other hand, it can be seen that by increasing the hyper-parameter b the power of NBF decreases, but by decreasing a the power of NBF method remains constant.

The effect of different hyper-parameter combinations was explored using 50

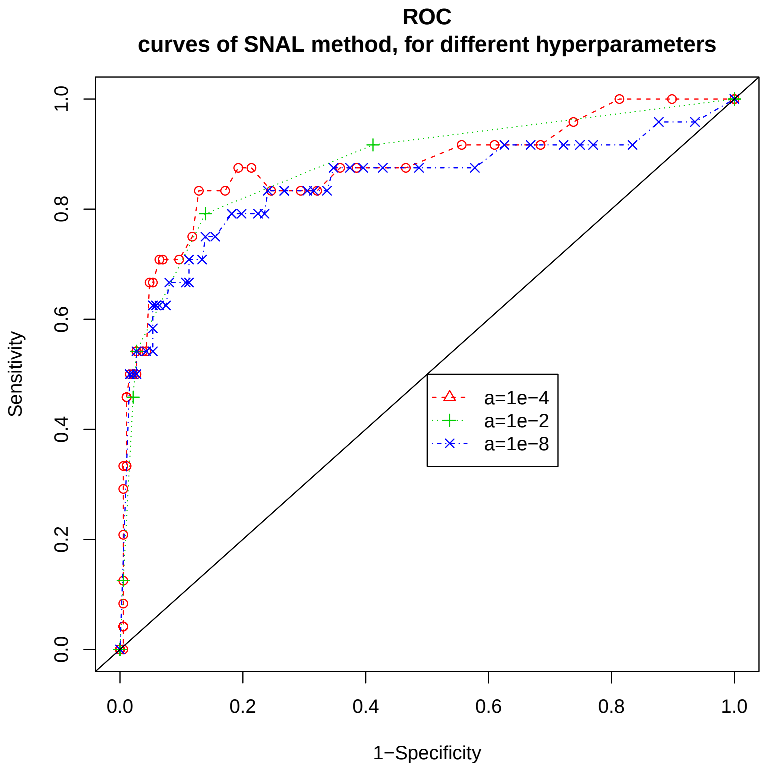

Figure 2: ROC curves of SNAL for different hyper-parameters. The AUC of the ROC curves for the three hyper-parameters are 0.8945, 0.8792 and 0.8679, respectively. The simulated response was created in the first case of the second scenario, ı.e the selected pathways were selected by SNAL.

simulated responses. The J associated pathways in this case are 40 randomly selected pathways.

Using paired t-tests the hyper-parameter combinations $(10^{-6}, 10^{-4}, 200, 0.25)$ and $(10^{-4}, 10^{-8}, 200, 0.25)$ have no statistical significant difference. Similarly the hyper-parameter combinations $(10^{-4}, 10^{-6}, 10, 0.10)$ and $(10^{-4}, 10^{-6}, 10, 2)$. On the other hand, the other hyper-parameter combinations are significantly different from each other (with p-values less than 0.05).

Moreover, there is no difference in the performance of SNAL when the hyper-parameter a equals $10^{-4}, 10^{-6}$ or 10^{-8}. On the other hand, the performance of the method is slightly lower when the hyper-parameter a is 10^{-2}.

Hyper-parameter Combination $(a, b, \nu_0, \sigma_0^2)$	Mean AUC	Median AUC
$(10^{-4}, 10^{-4}, 200, 0.25)$	0.5556 (0.0222)	0.5557 (0.0221)
$(10^{-6}, 10^{-6}, 200, 0.25)$	0.5873 (0.0330)	0.5851 (0.0341)
$(10^{-4}, 10^{-6}, 200, 0.25)$	0.5774 (0.0305)	0.5751(0.0308)
$(10^{-6}, 10^{-4}, 200, 0.25)$	0.5433 (0.0194)	0.5408 (0.0200)
$(10^{-4}, 10^{-8}, 200, 0.25)$	0.5422 (0.0278)	0.5451(0.0306)
$(10^{-4}, 10^{-6}, 10, 0.10)$	0.5714 (0.0293)	0.5713 (0.0308)
$(10^{-4}, 10^{-6}, 10, 2)$	0.5692 (0.0287)	0.5677 (0.0319)
$(10^{-4}, 10^{-6}, 200, 0.10)$	0.5815(0.0329)	0.5773 (0.0342)

Table 1: The effect of the hyper-parameters on the performance of NBF.

Hyper-parameter a	Mean AUC	Median AUC
10^{-2}	0.5729 (0.0334)	0.5724 (0.0299)
10^{-4}	0.5982 (0.0336)	0.5982 (0.0329)
10^{-6}	0.6008 (0.0322)	0.6047 (0.0321)
10^{-8}	0.6033 (0.0321)	0.6025 (0.0305)

Table 2: The effect of the hyper-parameter a on the performance of SNAL.

1.2 GWAS

Figure 3 shows the Residual vs Fitted values and the Q-Q Normal plot of equation (9). The response variable is the phenotype PC and the covariates matrix Φ includes only the J pathways that were found by NBF as associated with the PC phenotype. These pathways are given in Table 8. Both plots indicate that the data do follow a Normal distribution.

Similarly, Figure 4 shows the Residual vs Fitted values and the Q-Q Normal plot of equation (9). The response variable is BMI and the covariates matrix Φ includes only the J pathways that were found by SNAL as associated with BMI. These pathways are given in Table 12. Both plots indicate that the data do follow a Normal distribution.

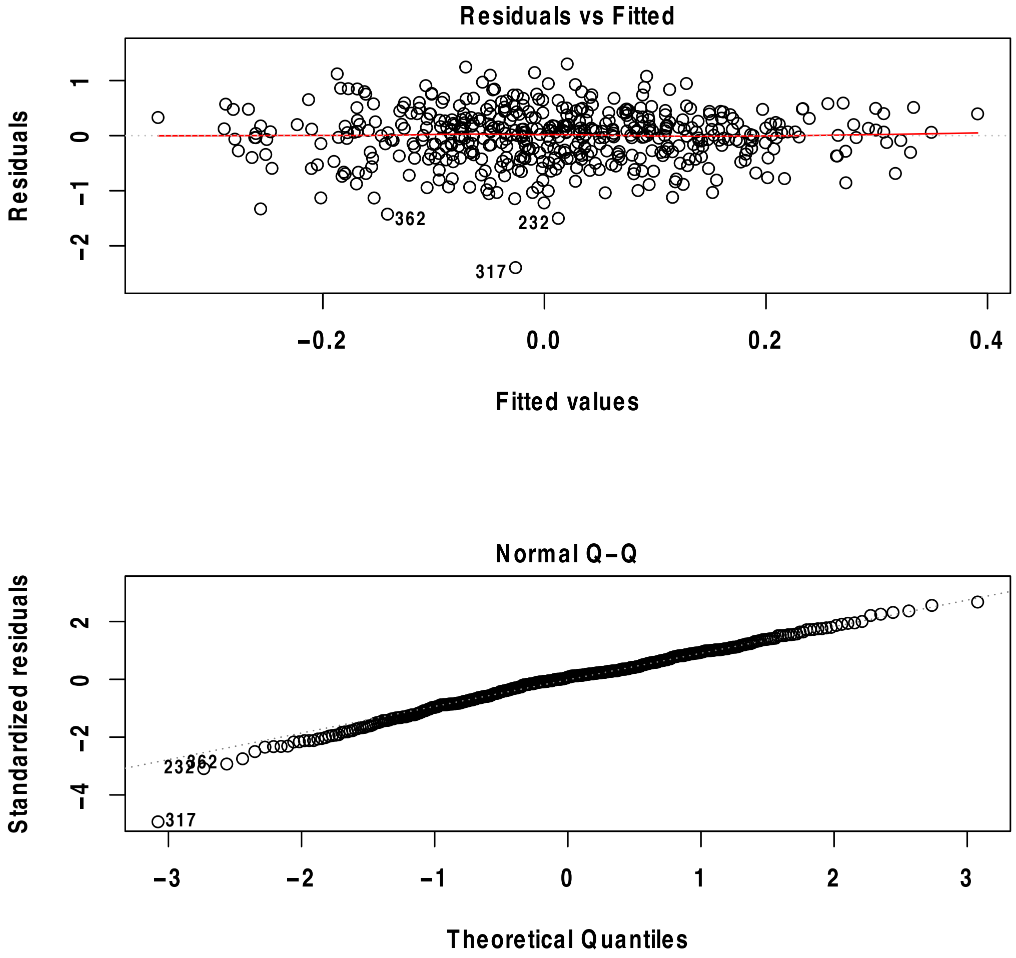

Figure 3: Residual vs Fitted values and the Q-Q Normal plot of the linear model of equation (9). The covariates matrix Φ includes only J pathways, that were found by NBF as associated with the PC phenotype. These pathways are given in Table 8.

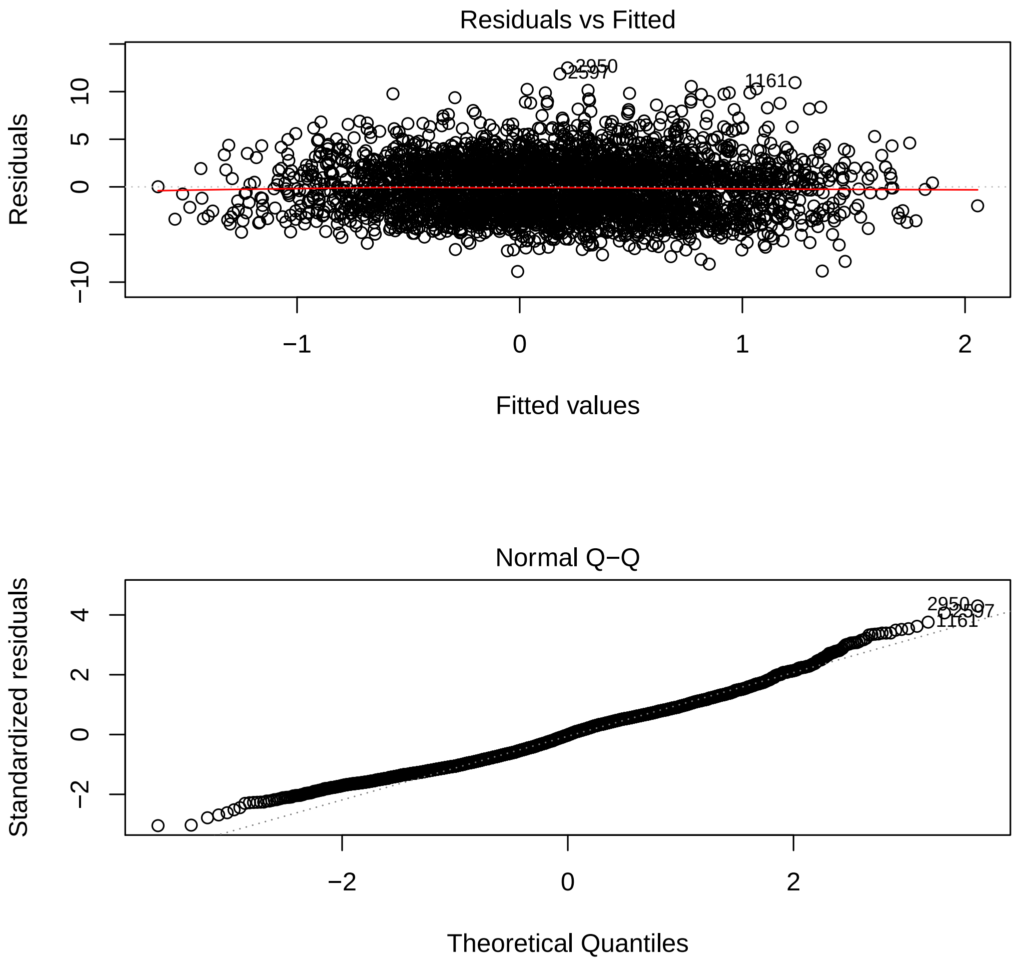

Figure 4: Residual vs Fitted values and the Q-Q Normal plot of the linear model of equation (9). The covariates matrix Φ includes only J pathways, that were found by SNAL as associated with BMI. These pathways are given in Table 12.

2 Simulation study

NBF	Method	Mean AUC	Median AUC
$(a, b) =$ $(10^{-2}, 10^{-2})$ $(\nu_0, \sigma_0^2) =$ $(10, 0.10)$	NBF<br>SNAL<br>FM<br>BGSA	0.8702 (0.0358)<br>0.8627 (0.0293)<br>0.6499 (0.0347)<br>0.5902 (0.0537)	0.8683 (0.0273)<br>0.8731 (0.0239)<br>0.6431 (0.0349)<br>0.5863 (0.0608)
$(a, b) =$ $(10^{-4}, 10^{-4})$ $(\nu_0, \sigma_0^2) =$ $(200, 2)$	NBF<br>SNAL<br>FM<br>BGSA	0.9593 (0.0291)<br>0.9150 (0.0309)<br>0.7276 (0.0570)<br>0.6239 (0.0572)	0.9684 (0.0231)<br>0.9212 (0.0201)<br>0.7288 (0.0593)<br>0.6093 (0.0525)

Table 3: Mean and median AUCs of the methods (with their standard deviations given in the brackets) for the two cases of scenario 1. The J selected pathways were selected by applying NBF to real data.

SNAL	Method	Mean AUC	Median AUC
$(a, \sigma^2) =$ $(10^{-2}, 0.05)$	NBF<br>SNAL<br>FM<br>BGSA	0.8613 (0.0308)<br>0.8761 (0.0352)<br>0.6449 (0.0351)<br>0.5734 (0.0602)	0.8632 (0.0337)<br>0.8777 (0.0363)<br>0.6467 (0.0335)<br>0.5697 (0.0856)
$(a, \sigma^2) =$ $(10^{-4}, 0.50)$	NBF<br>SNAL<br>FM<br>BGSA	0.9187 (0.0326)<br>0.9320 (0.0401)<br>0.7125 (0.0412)<br>0.6226 (0.0769)	0.9253 (0.0250)<br>0.9412 (0.0330)<br>0.7199 (0.0411)<br>0.6370 (0.0703)

Table 4: Mean and median AUCs (with their standard deviations given in the brackets) of the methods for the two cases of scenario 2. The J selected pathways were selected by applying SNAL to real data.

FM	Method	Mean AUC	Median AUC
p-value≤ 0.05	NBF<br>SNAL<br>FM<br>BGSA	0.5608 (0.0190)<br>0.5655 (0.0242)<br>0.6276 (0.0304)<br>0.5376 (0.0280)	0.5593 (0.0174)<br>0.5657 (0.236)<br>0.6288 (0.0275)<br>0.5296 (0.0262)
$p-$value$\leq \frac{0.05}{211}$	NBF<br>SNAL<br>FM<br>BGSA	0.5315 (0.0326)<br>0.5665 (0.0466)<br>0.7276 (0.0474)<br>0.6093 (0.0749)	0.5251 (0.0372)<br>0.5609 (0.0572)<br>0.7214 (0.0509)<br>0.6180 (0.0905)

Table 5: Mean and median AUCs (with their standard deviations given in the brackets) of the methods for the two cases of scenario 3. The J selected pathways correspond to the pathways that have FM p-values less than 0.05 and $\frac{0.05}{211}$, respectively.

BGSA	Method	Mean AUC	Median AUC
p-value≤ 0.05	NBF<br>SNAL<br>FM<br>BGSA	0.5949 (0.0286)<br>0.6148 (0.0445)<br>0.7587 (0.0550)<br>0.9099 (0.0906)	0.5958 (0.0258)<br>0.6226 (0.0417)<br>0.7669 (0.0666)<br>0.9378 (0.0649)
$p-$value$\leq \frac{0.05}{211}$	NBF<br>SNAL<br>FM<br>BGSA	0.6783 (0.0581)<br>0.7082 (0.0656)<br>0.7279 (0.0570)<br>0.8006 (0.1587)	0.6667 (0.0580)<br>0.7094 (0.0605)<br>0.7272 (0.0682)<br>0.8427 (0.1817)

Table 6: Mean and median AUCs (with their standard deviations given in the brackets) of the methods for the two cases of scenario 4. The J selected pathways correspond to the pathways that have BGSA p-values less than 0.05 and $\frac{0.05}{211}$, respectively.

2.1 Simulation study with binary data

The continuous response variable of the platelets GWAS was converted to a binary response and the performance of the methods in analysing binary data was tested. In total, 200 simulated phenotypes were computed.

Method	Mean AUC	Median AUC
NBF	0.6947 (0.0649)	0.7073 (0.0808)
SNAL	0.7136 (0.1145)	0.7348 (0.1182)
FM	0.6630 (0.0687)	0.6536 (0.0667)
BGSA	0.6260 (0.1233)	0.5793 (0.0790)

Table 7: Mean and median AUCs (with their standard deviations given in the brackets) of the methods for 200 simulated phenotypes converted to binary data.

Paired t-tests were used to test the difference of the four methods. SNAL is significantly superior to NBF. In addition, NBF is superior to the FM and BGSA method (with p-values less than 0.05).

Table 7 shows that the two proposed methods are powerful enough to be used for case-control data. It should be noted since the data of the platelets GWAS were used, the simulated case-control data included 240 cases and 240 controls, which is a not a representative number of a real GWAS, as most GWAS these days included thousands of individuals. We are expecting that the power of the two proposed methods, of the Fisher's method and of the BGSA method increases as the number of individuals increases.

3 Platelets GWAS

Method	Pathway name	BF	Number of SNPs
NBF	Glycosaminoglycan degradation	0.8809	404
	Glycosphingolipid biosynthesis	0.8743	572
	Endocytosis	0.8535	4213
	Phagosome	0.8199	2161
	Melanogenesis	0.8825	2125
	Salivary secretion	0.8243	3423
	Chagas disease	0.9195	1912
	Malaria	0.8991	628
	Renal cell carcinoma	0.9211	1173
	Basal cell carcinoma	0.9107	705
	Viral myocarditis	0.9378	1460
SNAL	Taurine and hypotaurine metabolism		69
	Glycosphingolipid biosynthesis		572
	Vitamin B6 metabolism		83
	Malaria		628
	Basal cell carcinoma		705

Table 8: KEGG pathways associated with PC phenotype as they are identified by NBF and SNAL.

Method	Pathway name	BF	Number of SNPs
NBF	Melanoma	0.8727	739
	Pentose phosphate pathway	0.9111	276
	Fructose and mannose metabolism	0.9272	684
	Galactose metabolism	0.9499	431
	Tyrosine metabolism	0.9337	560
	Mucin type O-Glycan biosynthesis	0.9021	1313
	Protein export	0.8358	378
	Calcium signaling pathway	0.9067	6547
	Apoptosis	0.8980	1099
	ECM-receptor interaction	0.9142	2590
	Tight junction	0.5065	4489
	Jak-STAT signaling pathway	0.9342	1703
	Leukocyte transendothelial migration	0.8384	2981
	Regulation of actin cytoskeleton	0.7216	4454
	Progesterone-mediated oocyte maturation	0.9264	1661
	Melanogenesis	0.9145	2125
	Vasopressin-regulated water reabsorption	0.9247	760
	Alzheimer's disease	0.7864	3272
	Pathways in cancer	0.9162	7541
	Glioma	0.9496	1400
	Melanoma	0.7040	1376
SNAL	Glycolysis/Gluconeogenesis		739
	Pentose phosphate pathway		276
	Vitamin B6 metabolism		83
	Tight junction		4489
	Alzheimer's disease		3272

Table 9: KEGG pathways associated with PA phenotype as they are identified by NBF and SNAL.

Method	Pathway name	BF	Number of SNPs
NBF	Arginine and proline metabolism	0.9470	509
	Glycosaminoglycan degradation	0.9277	404
	Glycerophospholipid metabolism	0.9026	1483
	Glycosphingolipid biosynthesis	0.9325	572
	PPAR signaling pathway	0.9425	1019
	MAPK signaling pathway	0.9052	5886
	Endocytosis	0.9111	4213
	Phagosome	0.9253	2161
	Hematopoietic cell lineage	0.9308	1296
	Long-term depression	0.9219	2916
	Melanogenesis	0.9284	2125
	Salivary secretion	0.6141	3423
	Chagas disease	0.7558	1912
	Renal cell carcinoma	0.8266	1173
	Basal cell carcinoma	0.9113	705
	Viral myocarditis	0.8676	1460
SNAL	Pentose phosphate pathway		276
	Glycosphingolipid biosynthesis		572
	Sulfur metabolism		283
	Salivary secretion		3423
	Renal cell carcinoma		1173
	Basal cell carcinoma		705

Table 10: KEGG pathways associated with FC phenotype as they are identified by NBF and SNAL.

Method	Pathway name	BF	Number of SNPs
NBF	Glycolysis/Gluconeogenesis	0.8717	739
	Mucin type O-Glycan biosynthesis	0.9172	1313
	PPAR signaling pathway	0.9150	1019
	Homologous recombination	0.9265	693
	Phosphatidylinositol signaling system	0.9469	2936
	p53 signaling pathway	0.8315	766
	Ubiquitin mediated proteolysis	0.9115	1853
	Notch signaling pathway	0.8377	764
	TGF-beta signaling pathway	0.9255	1114
	VEGF signaling pathway	0.9100	1203
	Focal adhesion	0.8972	5288
	Tight junction	0.9335	4489
	Jak-STAT signaling pathway	0.8791	1703
	Leukocyte transendothelial migration	0.774	2981
	GnRH signaling pathway	0.8574	2911
	Progesterone-mediated oocyte maturation	0.8157	1661
	Salivary secretion	0.8246	3423
	Parkinson's disease	0.7849	1439
	Pathways in cancer	0.9389	7541
	Basal cell carcinoma	0.9334	705
	Melanoma	0.9341	1376
SNAL	Glycolysis/Gluconeogenesis		739
	p53 signaling pathway		766
	Notch signaling pathway		764
	Parkinson's disease		1439
	Basal cell carcinoma		705

Table 11: KEGG pathways associated with FA phenotype as they are identified by NBF and SNAL.

4 EPIC-Norfolk GWAS

NBF	SNAL
Steroid biosynthesis	Glycolysis/Gluconeogenesis
Glycine, serine and threonine metabolism	Synthesis and degradation of ketone bodies
Inositol phosphate metabolism	Steroid biosynthesis
Porphyrin and chlorophyll metabolism	Glycine, serine and threonine metabolism
Proteasome	Lysine degradation
Cell cycle	Selenocompound metabolism
SNARE interactions in vesicular transport	Other glycan degradation
TGF-beta signaling pathway	Glycosaminoglycan biosynthesis - heparan sulfate
VEGF signaling pathway	Glycerolipid metabolism
Complement and coagulation cascades	Pyruvate metabolism
Jak-STAT signaling pathway	Thiamine metabolism
Hematopoietic cell lineage	Riboflavin metabolism
Fc epsilon RI signaling pathway	Vitamin B6 metabolism
Leukocyte transendothelial migration	Biotin metabolism
Melanogenesis	Folate biosynthesis
Amyotrophic lateral sclerosis (ALS)	Porphyrin and chlorophyll metabolism
Glioma	ABC transporters
Viral myocarditis	Proteasome
	PPAR signaling pathway
	Mismatch repair
	Non-homologous end-joining
	Cell cycle
	Oocyte meiosis
	SNARE interactions in vesicular transport
	Regulation of autophagy
	Lysosome
	Peroxisome
	Apoptosis
	Vascular smooth muscle contraction
	Hedgehog signaling pathway
	TGF-beta signaling pathway
	VEGF signaling pathway
	Cell adhesion molecules (CAMs)
	Complement and coagulation cascades
	Antigen processing and presentation
	NOD-like receptor signaling pathway
	Jak-STAT signaling pathway
	Hematopoietic cell lineage
	B cell receptor signaling pathway
	Fc epsilon RI signaling pathway
	Leukocyte transendothelial migration
	Circadian rhythm - mammal
	Phototransduction
	Melanogenesis
	Type II diabetes mellitus
	Aldosterone-regulated sodium reabsorption
	Collecting duct acid secretion
	Parkinson's disease
	Amyotrophic lateral sclerosis (ALS)
	Huntington's disease
	Bacterial invasion of epithelial cells
	Malaria
	Glioma
	Allograft rejection
	Viral myocarditis

Table 12: KEGG pathways associated with BMI as they are identified by NBF and SNAL.

NBF	SNAL
Steroid biosynthesis	Glycolysis/Gluconeogenesis
PPAR signaling pathway	Synthesis and degradation of ketone bodies
Mismatch repair	Steroid biosynthesis
ErbB signaling pathway	Glycine, serine and threonine metabolism
Cell cycle	Lysine degradation
Apoptosis	Selenocompound metabolism
TGF-beta signaling pathway	Other glycan degradation
Gap junction	Glycosaminoglycan biosynthesis -heparan sulfate
Complement and coagulation cascades	Glycerolipid metabolism
NOD-like receptor signaling pathway	Pyruvate metabolism
Jak-STAT signaling pathway	Thiamine metabolism
Hematopoietic cell lineage	Riboflavin metabolism
B cell receptor signaling pathway	Vitamin B6 metabolism
Melanogenesis	Biotin metabolism
Alzheimer's disease	Folate biosynthesis
Huntington's disease	Porphyrin and chlorophyll metabolism
Shigellosis	ABC transporters
	Proteasome
	PPAR signaling pathway
	Mismatch repair
	Non-homologous end-joining
	Cell cycle
	Oocyte meiosis
	SNARE interactions in vesicular transport
	Regulation of autophagy
	Lysosome
	Peroxisome
	Apoptosis
	Vascular smooth muscle contraction
	Hedgehog signaling pathway
	TGF-beta signaling pathway
	VEGF signaling pathway
	Cell adhesion molecules (CAMs)
	Complement and coagulation cascades
	Antigen processing and presentation
	NOD-like receptor signaling pathway
	Jak-STAT signaling pathway
	Hematopoietic cell lineage
	B cell receptor signaling pathway
	Fc epsilon RI signaling pathway
	Leukocyte transendothelial migration
	Circadian rhythm - mammal
	Phototransduction
	Melanogenesis
	Type II diabete mellitus
	Aldosterone-regulated sodium reabsorption
	Collecting duct acid secretion
	Parkinson's disease
	Amyotrophic lateral sclerosis (ALS)
	Huntington's disease
	Bacterial invasion of epithelial cells
	Malaria
	Glioma
	Allograft rejection
	Viral myocarditis

Table 13: Pathways associated with binary BMI identified by NBF and SNAL. The KEGG pathways have BF<=0.765.
